# Monte Carlo simulation of beam characteristics from small fields based on TrueBeam flattening-filter-free mode

**DOI:** 10.1186/s13014-016-0601-2

**Published:** 2016-02-27

**Authors:** Zhongsu Feng, Haizhen Yue, Yibao Zhang, Hao Wu, Jinsheng Cheng, Xu Su

**Affiliations:** Key laboratory of Radiological Protection and Nuclear Emergency, Chinese Center for Disease Control and Prevention, National Institute for Radiological Protection, Beijing, 100088 China; Key laboratory of Carcinogenesis and Translational Research (Ministry of Education/Beijing), Department of Radiotherapy, Peking University Cancer Hospital & Institute, 52 Fuchen Road, Haidian, Beijing, 100142 China

**Keywords:** TrueBeam, Accelerator, Flattening-filter-free, Monte Carlo, Small field, Percentage depth dose, Off-axis ratio

## Abstract

**Purpose:**

Through the Monte Carlo (MC) simulation of 6 and 10 MV flattening-filter-free (FFF) beams from Varian TrueBeam accelerator, this study aims to find the best incident electron distribution for further studying the small field characteristics of these beams.

**Methods:**

By incorporating the training materials of Varian on the geometry and material parameters of TrueBeam Linac head, the 6 and 10 MV FFF beams were modelled using the BEAMnrc and DOSXYZnrc codes, where the percentage depth doses (PDDs) and the off-axis ratios (OARs) curves of fields ranging from 4 × 4 to 40 × 40 cm^2^ were simulated for both energies by adjusting the incident beam energy, radial intensity distribution and angular spread, respectively. The beam quality and relative output factor (ROF) were calculated. The simulations and measurements were compared using Gamma analysis method provided by Verisoft program (PTW, Freiburg, Germany), based on which the optimal MC model input parameters were selected and were further used to investigate the beam characteristics of small fields.

**Results:**

The Full Width Half Maximum (FWHM), mono-energetic energy and angular spread of the resultant incident Gaussian radial intensity electron distribution were 0.75 mm, 6.1 MeV and 0.9° for the nominal 6 MV FFF beam, and 0.7 mm, 10.8 MeV and 0.3° for the nominal 10 MV FFF beam respectively. The simulation was mostly comparable to the measurement. Gamma criteria of 1 mm/1 % (local dose) can be met by all PDDs of fields larger than 1 × 1 cm^2^, and by all OARs of no larger than 20 × 20 cm^2^, otherwise criteria of 1 mm/2 % can be fulfilled. Our MC simulated ROFs agreed well with the measured ROFs of various field sizes (the discrepancies were less than 1 %), except for the 1 × 1 cm^2^ field.

**Conclusions:**

The MC simulation agrees well with the measurement and the proposed model parameters can be clinically used for further dosimetric studies of 6 and 10 MV FFF beams.

## Introduction

To facilitate the model development and dose computation, conventional radiation beam was flattened through the filter mounted in the gantry head. However, the application of intensity-modulated radiation therapy (IMRT) and volumetric-modulated arc radiotherapy (VMAT) techniques have made the uniform beam less necessary. In addition, the flattening filter free (FFF) beam provides much higher dose rate and less head scattering [1–3], which has been increasingly applied to the stereotactic body radiotherapy (SBRT) and stereotactic radio-surgery (SRS) for better delivery efficiency [4–6]. Clinically speaking, tumour size of less than 5 cm in diameter is usually considered as suitable for SBRT and SRS, yet it is more challenging for the dosimetric measurement with a decreasing field size [7]. Due to the disequilibrium of charged particles and ionization chamber volume averaging effects, the measurement uncertainty of the central axis depth dose and beam profiles for small fields may severely undermine the accuracy of clinical dosimetry. Alternatively, the Monte Carlo (MC) method provides accurate simulation of the machine geometry and particle interactions [7–9], which was used to investigate the small field dosimetry of FFF beams in this study.

TrueBeam accelerator (Varian Medical Systems, Palo Alto, CA) is capable of generating 6 and 10 MV FFF photon beams. Accurate geometric and material parameters of the Linac head are critical for the MC modelling, yet they have not been made available for TrueBeam except for the first and second generation phase-space files. The first generation phase-space was created on a cylindrical surface, and Constantin et al. [10] converted the format of these phase-space files before performing validations in a water phantom for various field sizes ranging from 1 × 1 to 40 × 40 cm^2^. The second generation phase-space was tallied right above the secondary jaws. Belosi et al. [11] evaluated the accuracy of the distributed phase-space files for FFF beams by comparing them with experimental measurements based on ten TrueBeam systems, and concluded that although the phase-space files can be used for accurate MC dose estimation, their applications to MC simulation were limited. As a solution, Rodriguez et al. [12] replaced the standard flattening filter (FF) with ad hoc thin filters that were modelled by comparing the dose measurements and simulations, and further analyzed the geometry validation of the FakeBeam. Relative to the Varian phase-space files, the ansatz geometry reproduced the measured dose more accurately, but the thin filters were made of high Z materials that increased the head scatter and affected the beam quality.

In this study, we selected appropriate material and geometry of the target and foil for 6 and 10 MV FFF beams of TrueBeam Linac based on the training materials of Varian. The other structures were consistent with Varian iX Linacs that have been released for research before. Based on these physical models, the BEAMnrc and DOSXYZnrc codes [13, 14] were used to simulate the percentage depth doses (PDDs) and the off-axis ratios (OARs) curves for 6 and 10 MV FFF X-ray with field sizes ranging from 4 × 4 to 40 × 40 cm^2^. The incident beam energy, radial intensity distribution and angular spread were adjusted respectively to get the optimum parameters for the model, which were used to investigate the characteristics of small fields of less than 4 × 4 cm^2^.

## Materials and methods

### Measurements

The measured data were acquired during the commissioning of TrueBeam. Based on a water tank with a scanning range of 60 × 50 × 40.8 cm^3^ (PTW, Freiburg, Germany), the lateral profiles and central axis depth doses were measured with different square fields from 1 × 1 to 40 × 40 cm^2^ at source to surface distance (SSD) equal to 100 cm. The measurements were conducted at various depths (d_max_, 5, 10, 20 and 30 cm respectively) in water with Diode P (PTW, Freiburg, Germany, Type 60016) detector. The central axis depth doses for fields of no larger than 4 × 4 cm^2^ were also measured using the Diode P detector; otherwise Roos plane parallel chamber (PTW, Freiburg, Germany) was used instead. For the penumbra region of OARs, and for the PDDs from the surface to 15 cm in depth, the measurement step interval was 1 mm, otherwise the distance was 2 mm/5 mm. In order to demonstrate a smooth transition between the measurement sets, PDDs of 4 × 4 cm^2^ field size were measured with both detectors respectively.

### Monte Carlo codes and parameters

Using BEAMnrc and DOSXYZnrc user codes, the MC simulations were performed based on a System X3850 X5 server consisting of 160 Intel Xeon central processing units (2.0 GHz each) and RAM of 256 GB.

According to the training materials of Varian, 6 and 10 MV FFF beams were generated by the TrueBeam accelerators by replacing the flattening filters mounted on the carousel port with thin brass foils. The 6 MV FFF beam uses the same low energy target for the flattened 6 X energy mode, whose parameters have been released. Different from the medium energy target that is used for generating the flattened 10 X beam, the 10 MV FFF beam uses high energy target whose data have not been made available by far. The trial simulation of the PDDs under the field size of 6 × 6 cm^2^ using the parameters of the 15 MV, 18 MV and 20 MV high energy targets did not agree well with the measurement of 10 MV FFF beam. Therefore, the 10 MV FFF beam target geometry and material (Tungsten and Copper) composition parameters were fine-tuned. The other structures were consistent with Varian iX Linacs that have been released before.

The BEAMnrc source (isource = 9: BEAM Treatment Head Simulation Incident from Any Direction) [14] was used as simulation source, which is similar to the isource = 2 (full phase-space file) but does not need to store a phase-space file.

The photon and electron cut off energy (PCUT and ECUT) values were set to 0.01 and 0.521 MeV, respectively. EXACT was selected as the electron step and boundary crossing algorithm. These settings were applied to both DOSXYZnrc and BEAMnrc user codes. The variance reduction technique of directional bremsstrahlung splitting (DBS) [13, 15] was used to increase computational efficiency. The radius of the smallest tangent circle to the entire treatment field was chosen as the splitting radius; therefore the contribution of fat photons in the region of interest was negligible. The bremsstrahlung splitting number (NBRSPL) was set to 1000 [15] for the maximum photon fluence efficiency.

The primary electron was set to 1 × 10^9^ histories in BEAMnrc code. DOSXYZnrc was used to perform all dose calculations, and 5 × 10^9^–1 × 10^10^ histories were simulated. The number of histories was adjusted for each field size to achieve the MC uncertainty < 0.5 %. The MC uncertainty is the average of the statistical uncertainties of all dose values in the dose distribution with more than 50 % of its maximum dose.

The water tank phantom of 50 × 50 × 50 cm^3^ in size was simulated. Source particles were scored at *SSD* = 100 cm. These BEAMnrc sources were inputted to DOSXYZnrc to obtain the central axis depth doses and the beam profiles in water of various depths and field sizes. The voxel size of 2 × 2 × 1 (x × y × z) mm^3^ was used to calculate the PDDs on the central axis. The voxel size of 0.5 × 1 × 1 mm^3^ was used to calculate the OARs for the field size of smaller than 3 × 3 cm^2^, otherwise the voxel size of 1 × 1 × 1 mm^3^ was used.

Combined with the aforementioned model parameters, the incident beam energy, radial intensity distribution and angular spread were adjusted respectively for better agreement between the simulated and measured PDDs and OARs of different field sizes. And then, the optimum parameters of the incident electron were used to investigate the characteristics of small fields of less than 4 × 4 cm^2^.

### Data comparison and analysis

The MATLAB software was used to extract the simulated data. Using VeriSoft software (version 5.1) (PTW, Freiburg, Germany), the gamma evaluation [16] was performed to compare the measured data (PDDs and OARs) with the simulated data. Passing criterion was met if the gamma index was no larger than 1.

The beam quality was specified by the tissue phantom ratio *TPR*_20,10_ [17] defined as:

1 $$ TP{R}_{20,10}=1.2661PD{D}_{20,10}-0.0595, $$

where PDD_20, 10_ is the ratio of the percent depth doses at 20 and 10 cm depths for the field size of 10 × 10 cm^2^ defined at the phantom surface with an SSD of 100 cm.

The formula for the relative output factor (ROF) of Popescu et al. [18] was used in our MC simulation. The penumbras analysis is based on the article by Fogliata et al. [19]. Before penumbras analysis, the FFF photon profile will be renormalized according to the requirements set forth in the article. The penumbra is the distance between the positions of the 80 and 20 % dose values of the renormalized profile.

## Results and discussion

The MC simulated results agreed well with the measurements. For all simulations, the average of the statistical uncertainties of all dose values was between 0.1 and 0.5 % for the dose distribution with more than 50 % of its maximum dose. For sake of clarity, they are not shown in figures.

The Full Width Half Maximum (FWHM), mono-energetic energy, and angular spread of the resultant incident Gaussian radial intensity electron distribution were 0.75 mm, 6.1 MeV and 0.9° respectively for the nominal 6 MV FFF beam, and were 0.7 mm, 10.8 MeV and 0.3° respectively for the 10 MV FFF beam.

The beam qualities of 6 and 10 MV FFF beams are listed in Table 1. The measured beam qualities are consistent with the results of Fogliata et al. [19]. The differences between the simulated and measured beam qualities were less than 0.5 % for both beams.

**Table 1:** The beam qualities of 6 and 10 MV FFF beams

Beam	TPR_20, 10_		
	Simulated	Measured	%Diff
6 FFF	0.628 ± 0.001	0.630	−0.29 ± 0.15
10 FFF	0.705 ± 0.001	0.707	−0.28 ± 0.14

As shown in Table 2, the disparities between the simulated and measured profile penumbras of various field sizes at 10 cm depth were within 1 mm for all field sizes. The calculated penumbra was pronounced smaller than the measured result when the field was less than 4 × 4 cm^2^. A possible reason of that difference might be partly ascribed to the difference of the lateral voxel resolution in the penumbra region between the MC calculations (0.5 mm) and the measurements (1 mm of step interval).

**Table 2:** The simulated and measured profiles penumbras (mm) of various field sizes at 10 cm depth

Field size (cm^2^)	6 FFF			10 FFF		
	Measured	Simulated	Diff.	Measured	Simulated	Diff.
1 × 1	2.4	2.0	0.4	3.2	2.7	0.5
2 × 2	2.6	2.2	0.4	3.6	3.3	0.3
3 × 3	2.7	2.4	0.3	3.8	3.4	0.4
4 × 4	2.9	2.8	0.1	3.9	3.7	0.2
6 × 6	3.2	3.0	0.2	4.1	4.0	0.1
10 × 10	3.6	3.6	0.0	4.5	4.5	0.0
20 × 20	5.1	5.1	0.0	5.4	5.4	0.0
40 × 40	8.3	8.6	−0.3	6.6	7.0	−0.4

**Fig. 1:**
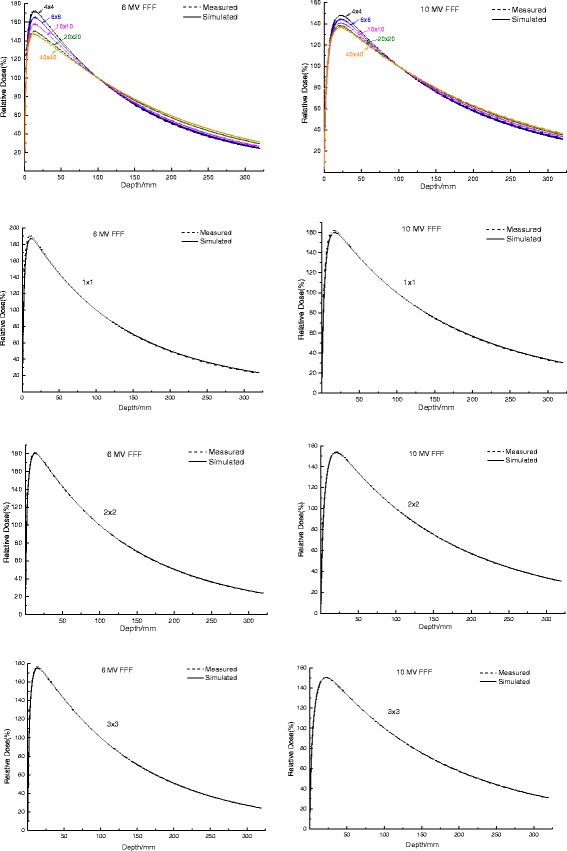
Measured and MC calculated PDDs for various field sizes (cm^2^) of 6 and 10 MV FFF beams. Statistical uncertainties of all dose values are between 0.1 and 0.5 % and are not shown

**Fig. 2:**
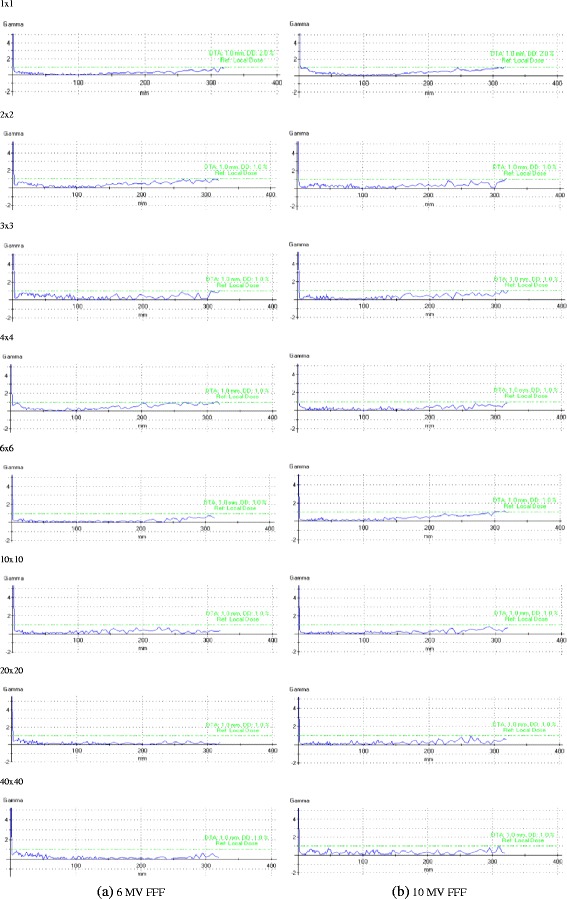
The gamma analysis results of PDDs for various field sizes (cm^2^) of 6 and 10 MV FFF beams. (DTA: Distance-To-Agreement, DD: Dose Difference)

The measured and MC calculated PDDs for various field sizes of 6 and 10 MV FFF beams were plotted in Fig. 1. All depth dose curves were normalized to 10 cm in depth. The gamma analyses for the comparisons of PDDs are shown in Fig. 2. For the depths from 0.1 cm to 30 cm, Gamma criteria of 1 mm/1 % (local dose) can be met by all PDDs for the fields of larger than 1 × 1 cm^2^ in size. For the field size of 1 × 1 cm^2^, the criteria of 1 mm/2 % can be fulfilled. Meanwhile, as shown in Table 3, the MC simulated ROFs agreed well with the measured ROFs of various field sizes (the discrepancies were less than 1 %), except for the 1 × 1 cm^2^ field. There were several possible reasons accountable for these. The first one was the lateral charged particle disequilibrium. The lack of lateral electron equilibrium and the volume averaging of the detector induced the reduced signal observed in the central part of the beam and a drop in the measured beam output [7]. Another important reason was the Linac gantry sag caused by the gravity (0.7 mm for our TrueBeam accelerator), due to which the detector was misaligned with the radiation centre during the measurement of PDDs and could considerably impact the results of small fields, especially in the 1 × 1 cm^2^ field size, and depth over 15 cm. Thirdly, the over-shielding of the Diode P detector which we used to measure the PDDs of field sizes less than 4 × 4 cm^2^ may have some impact on the results.

**Table 3:** The simulated and measured relative output factors of various field sizes

Field size (cm^2^)	6 FFF			10 FFF		
	Measured	Simulated	%Diff.	Measured	Simulated	%Diff.
1 × 1	0.684	0.717	4.82	0.697	0.721	3.40
2 × 2	0.801	0.807	0.80	0.845	0.848	0.34
3 × 3	0.842	0.849	0.82	0.894	0.895	0.10
4 × 4	0.875	0.873	−0.17	0.921	0.926	0.52
6 × 6	0.929	0.932	0.25	0.956	0.962	0.66
10 × 10	1.000	1.000	—	1.000	1.000	—
20 × 20	1.080	1.073	−0.66	1.049	1.045	−0.36
40 × 40	1.122	1.117	−0.47	1.069	1.063	−0.58

**Fig. 3:**
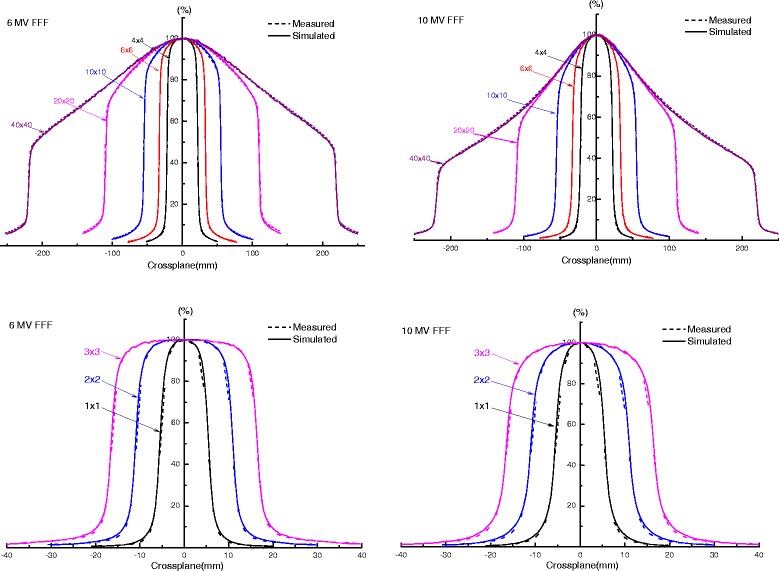
Measured and MC calculated off-axis dose profiles for various field sizes (cm^2^) at 10 cm depth of 6 and 10 MV FFF beams. Statistical uncertainties of all dose values are between 0.1 and 0.5 % and are not shown

**Fig. 4:**
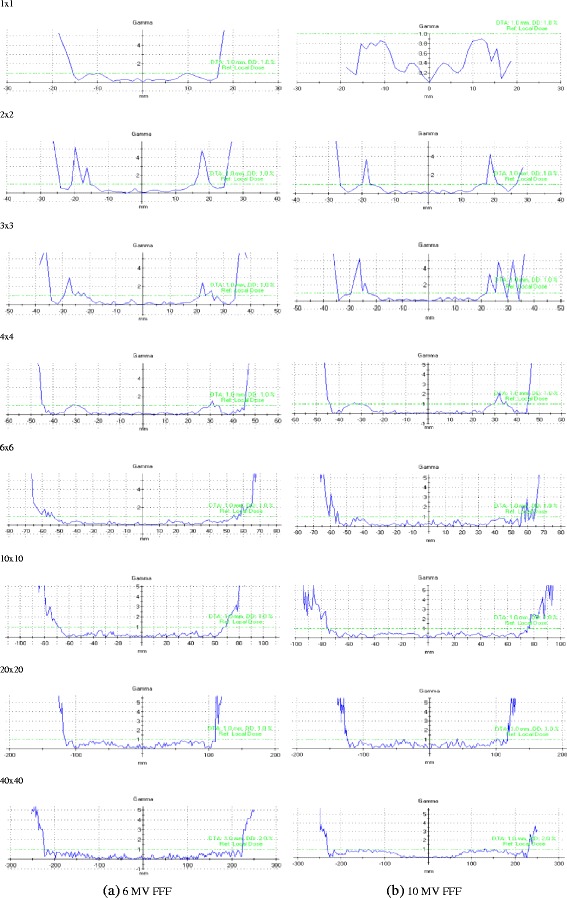
The gamma analysis results of OARs for various field sizes (cm^2^) at 10 cm depth of 6 and 10 MV FFF beams. (DTA: Distance-To-Agreement, DD: Dose Difference)

**Fig. 5:**
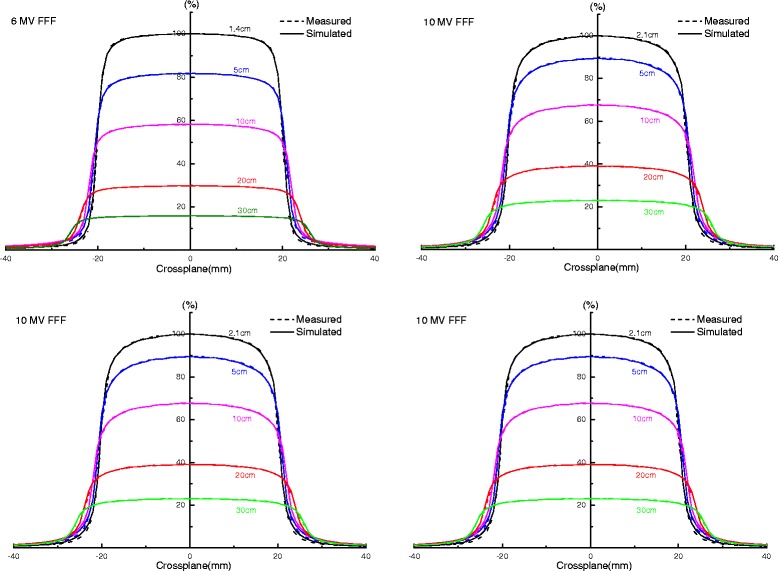
The comparison of measured and MC calculated off-axis dose profiles for 4 × 4 and 10 × 10 cm^2^ field sizes (cm^2^) at different depth (d_max_, 5, 10, 20 and 30 cm) of 6 and 10 MV FFF beams. Statistical uncertainties of all dose values are between 0.1 and 0.5 % and are not shown

The measured and MC calculated off-axis dose profiles for various field sizes at 10 cm depth are shown in Fig. 3. All profiles were normalized to 100 % on the central axis for all beam modes. The gamma analysis results of OARs for various field sizes at 10 cm depth are shown in Fig. 4. The agreement was within 1 mm/1 % for field sizes of less than 30 × 30 cm^2^, within 1 mm/2 % for other field sizes of 6 MV FFF beams, within 1 mm/1 % for field sizes of less than 20 × 20 cm^2^ for 10 MV FFF beams, and within 1 mm/2 % for the other field sizes respectively. The comparison of the measured and MC calculated off-axis dose profiles for 4 × 4 and 10 × 10 cm^2^ field sizes at different depths (d_max_, 5, 10, 20, and 30 cm) are shown in Fig. 5. The comparison of the lateral dose profiles showed that the simulation reproduced the measurement well, especially for the fields of less than 30 × 30 cm^2^. Our results based on field sizes of less than 10 × 10 cm^2^ are more clinically relevant to the actual application of FFF beams than previous studies [10–12]. The MC calculated OARs for the inline direction were also in good agreement with the measured results which are also not shown in Figures.

Regarding the limitations of this study, the accuracy and appropriateness of modelling the target of 10 MV FFF can be questioned, which is crucial for the beam quality. But the proposed model agreed well with the measured data. Another limitation is the unknown geometry of the monitor ionization chamber. The actual structure of the monitor ionization chamber is more complex than the ionization chamber model used in this study, which may affect the accuracy of the MC absolute dosimetry. Since the geometric data of the Linac head is still unknown, the proposed modelling method in this study is a reasonable approximation for the MC simulation of the FFF Beams. Additionally, it should be noted that although the measured data were used as a reference value, it was also subject to some uncertainties especially for small fields due to the detector properties and the mechanical/physical properties of the Linac. Therefore, we approximated the optimum model parameters based on the larger field, and investigated the small field dosimetry of less than 4 × 4 cm^2^ based on these parameters. In addition, the dose distributions were normalized individually before comparison in this study, hence the goodness of the gamma evaluation might be overestimated than in other studies using absolute dose distributions.

## Conclusions

Using the proposed model parameters in this study, the MC simulated results agreed well with the measurements hence can be used for further clinical dosimetric studies involving 6 and 10 MV FFF X-ray. Although the head model used in this study can approximate the beam data, the actual structural information of the TrueBeam accelerator is necessary to verify the accuracy of these model parameters. Further studies are needed for a complete investigation of the characters of FFF beams especially for the small field sizes.

## Abbreviations

DBS: directional bremsstrahlung splitting
FFF: flattening-filter-free
FF: flattening filter
FWHM: full width half maximum
IMRT: intensity-modulated radiation therapy
MC: Monte Carlo
OARs: off-axis ratios
PCUT and ECUT: photon and electron cut off energy
PDDs: percentage depth doses
ROF: relative output factor
SBRT: stereotactic body radiotherapy
SRS: stereotactic radio-surgery
SSD: source to surface distance
TPR: tissue phantom ratio
VMAT: volumetric-modulated arc radiotherapy
